# Targeted repositioning identifies drugs that increase fibroblast growth factor 20 production and protect against 6-hydroxydopamine-induced nigral cell loss in rats

**DOI:** 10.1038/s41598-019-44803-1

**Published:** 2019-06-06

**Authors:** Edward J. R. Fletcher, Aran D. Jamieson, Gareth Williams, Patrick Doherty, Susan Duty

**Affiliations:** King’s College London, Institute of Psychiatry, Psychology & Neuroscience, Wolfson Centre for Age-Related Diseases, Guy’s Campus, London, SE1 1UL UK

**Keywords:** Parkinson's disease, Neurotrophic factors

## Abstract

Endogenous fibroblast growth factor 20 (FGF20) supports maintenance of dopaminergic neurones within the nigrostriatal pathway. Moreover, direct intracerebral infusion of FGF20 protects against nigrostriatal tract loss in the 6-hydroxydopamine lesion rat model of Parkinson’s disease. Increasing endogenous FGF20 production might provide a less-invasive, more translational way of providing such protection. Accordingly, we adopted a *targeted repositioning* approach to screen for candidate FDA-approved drugs with potential to enhance endogenous FGF20 production in brain. *In silico* interrogation of the Broad Institute’s Connectivity Map database (CMap), revealed 50 candidate drugs predicted to increase FGF20 transcription, 16 of which had profiles favourable for use in Parkinson’s disease. Of these, 11 drugs were found to significantly elevate FGF20 protein production in MCF-7 cells, between two- and four-fold. Four drugs were selected for examination *in vivo*. Following oral dosing in rats for 7 days, salbutamol and triflusal, but not dimethadione or trazodone, significantly elevated FGF20 levels in the nigrostriatal tract. Preliminary examination in the unilateral 6-hydroxydopamine-lesioned rat revealed a modest but significant protection against nigral cell loss with both drugs. Our data demonstrate the power of *targeted repositioning* as a method to identify existing drugs that may combat disease progression in Parkinson’s by boosting FGF20 levels.

## Introduction

Parkinson’s disease (PD) is a neurodegenerative disorder characterised by motor symptoms such as tremor, bradykinesia and postural instability^1^. These symptoms largely result from degeneration of dopaminergic neurones in the substantia nigra pars compacta (SNc). Current pharmacological treatments, which mostly replenish the lost dopamine (e.g. L-DOPA), provide some relief of the motor symptoms. However, these treatments do not combat the underlying dopaminergic neurodegeneration, so the disease pathology and associated symptoms progress and remain a significant problem. There remains a clear need for treatments that might slow or halt disease progression. Given the pathogenesis of PD is wide-ranging^2^ and syndrome-like^3^, interfering with individual mechanisms has yet to yield success. A more fruitful approach to providing neuroprotection might be to use neurotrophic factors to enhance the survival of the vulnerable neurones exposed to such multiple insults.

Fibroblast growth factor 20 (FGF20) is a possible candidate for providing neuroprotection in PD. FGF20 is a brain-specific factor that is abundant in the nigrostriatal pathway where it plays a role in dopamine neurone development, maintenance and survival^4,5^. Addition of exogenous FGF20 has been shown to protect cultured dopaminergic neurones from toxic insults including glutamate and the dopaminergic toxin, 6-hydroxydopamine (6-OHDA)^4,6^. Furthermore, we found that supra-nigral infusion of recombinant human FGF20 (rhFGF20) protected against dopaminergic neurone degeneration and preserved motor function in both full and partial 6-OHDA lesion rat models of PD^6,7^.

While the approach of using neurotrophic factors therapeutically in PD has maintained traction, the failure to demonstrate efficacy in clinical trials is disappointing^8,9^. Clinical progress may have been hampered due to the reliance on direct intracerebral delivery, insufficient distribution and the targeting of a single site - dopaminergic terminals in the striatum – from where retrograde transport back to the substantia nigra may be limited by impaired axonal transport and signalling in PD^10–13^. Finding alternative ways of increasing trophic factor levels in the brain might overcome these hurdles. A recent study demonstrated that ultrasonography-guided rhFGF20 proteoliposomes can provide protection in the 6-OHDA lesioned rat when delivered systemically, avoiding the need for direct intracerebral delivery^14^. As an alternative approach, we explored the possibility of therapeutically driving *endogenous* FGF20 production rather than relying on direct infusion of an exogenous supply.

We recently identified GFAP-positive astrocytes as a source of FGF20 within the substantia nigra^7^. Given these cells are spared in PD^15,16^, they provide a potential source from which to boost production of endogenous FGF20. In order to find suitable drugs to achieve this, we have undertaken a novel, targeted repositioning approach using a combination of bioinformatics, *in vitro* and *in vivo* assays. Specifically, we interrogated the transcriptional profiles of more than a thousand Food and Drug Administration (FDA)-approved drugs from the Broad Institute’s connectivity mapping database^17^ to identify drugs that increase FGF20 gene transcription. We selected those that cross the blood-brain barrier and have no contra-indication for use in PD, and screened for their ability to boost endogenous FGF20 protein production *in vitro*. A sub-set of the most promising drugs progressed through to *in vivo* studies to determine FGF20 production in relevant brain regions. Finally, we explored the protective efficacy of the best two drugs in a preliminary study in the partial 6-OHDA lesion rat model of PD, to generate proof of concept for our targeted repositioning approach. This approach revealed salbutamol and triflusal as the two most promising drugs of interest.

## Material and Methods

### Bioinformatics screening to draw up a shortlist of potential FGF20 boosting drugs

*In silico* screening involved interrogation of the connectivity map (CMap) from the Broad Institute^17^ for drugs that increase FGF20 transcription. The CMap consists of the gene expression profiles gathered from three human cancer cell lines (MCF-7, PC3 and HL60) for 1261 drug-like compounds. Robust profiles were defined as previously described^18^. Briefly, the gene expression change ranks, defined as $=1-2\frac{R-{R}_{min}}{{R}_{max}-{R}_{min}}$, where *R* is the rank of a given gene’s expression change (*R*_*max*_ being the highest and *R*_*min*_ being the lowest ranks), were averaged over replicates, ignoring cell type, and filtered based on significance using a one sample student’s t-test. Drug candidates for the up-regulation of FGF20 were ranked based on the average expression rank of FGF20 in the given drug’s CMAP profile. The top 50 ranking compounds were subject to further literature-based scrutiny to rule out drugs with low blood-brain barrier (BBB) penetration probability, with anticipated contraindications for use in PD or with the known emergence of toxicity following chronic dosing.

### Assessment of FGF20 production in MCF-7 cells or ventral mesencephalic (VM) primary cultures following treatment with selected drugs

MCF-7 human breast carcinoma cells (Sigma) were utilised for the initial drug screen to maintain consistency with the cells used to generate the transcriptional profiles in the CMap database. MCF-7 cells were maintained in DMEM-Glutamax media with 10% foetal bovine serum (FBS), 100 μg/ml streptomycin and 100 units/ml penicillin (1% penstrep, Gibco) at 37 °C in 5% CO_2_. Cells were incubated (~250,000 viable cells per well) in a 6-well plate for 24 h at 37 °C in 5% CO_2_. Cells were then incubated in FBS-free DMEM-Glutamax medium containing 10 µM of candidate drug for a further 24 h. This concentration was chosen for consistency with that used for the transcriptional profiling. Each drug and respective vehicle was tested on a minimum of three independent cultures. After washing with phosphate-buffered saline (PBS), cells were detached with 0.25% trypsin for 5 min at 37 °C before lysing by freeze-thawing and high-frequency sonication in lysis buffer. After centrifugation (10,000 rpm for 5 min at 4 °C), sample lysates were diluted in dH_2_0 to 1 mg/ml protein using a standard bicinchoninic acid (BCA) assay, in preparation for FGF20 quantification.

E13.5 timed-pregnant female Sprague-Dawley rats (Envigo; n = 3) were killed with an overdose of sodium pentobarbital (200 mg, i.p.) and cervical dislocation and the embryos (10–15 per litter) removed. Ventral mesencephalic (VM) brain tissue was dissected out, pooled for a given litter and washed thrice in ice-cold PBS before incubating in 1 ml 0.25% trypsin in PBS at 37 °C for 10 min. 9 ml of DMEM Glutamax media with 10% FBS and 1% penstrep was added and the cell suspension centrifuged for 5 min at 5000 rpm. The pellet was resuspended in 1 ml of fresh medium and triturated to achieve a single cell suspension. Trypan blue excluding, viable cells were plated (300,000 cells per coverslip) onto sterile 13 mm diameter poly-D-lysine coated glass coverslips within a 24-well plate. Cultures were incubated in 10% FBS-containing medium at 37 °C in a humidified atmosphere of 5% CO_2_. At 3 days *in-vitro* (DIV3), media was aspirated and fresh media added. At 6 days *in-vitro* (DIV6), cells were washed twice with PBS then incubated for 24 h with either vehicle or one of five drugs of choice diluted in FBS-free medium before cells were lysed as described above. Given that the VM cultures were used to confirm FGF20 production in a physiologically-relevant cell system, but the effective concentrations would not help inform the doses selected for *in-vivo* studies, we again examined each drug at a single concentration, consistent with that used in the MCF-7 cells (10 µM).

Total FGF20 content within the MCF-7 and VM cell lysates was measured by sandwich enzyme-linked immunosorbent assay (ELISA) kits (Cusabio, CSB-EL008626HU). Samples were processed, in duplicate, according to the supplier’s protocol. ELISAs were repeated (three technical replicates) to ensure reliable findings.

### Animals

All *in-vivo* studies were performed in accordance with the UK Animals Scientific Procedures Act (1986) and were approved by King’s College London Animal Welfare and Ethical Review Body. A total of 120 adult male Sprague Dawley rats (250–280 g; Charles River, UK) were used: 80 for the *in-vivo* screening and 40 for the 6-OHDA neuroprotection study. All animals were maintained on a 12:12 hour light:dark cycle with food and water available *ad libitum*.

### Assessment of FGF20 production in brain regions of interest in rats treated with selected drugs

Four drugs were taken through to the *in vivo* examination: dimethadione, salbutamol, trazodone and triflusal. For each drug study, adult male Sprague Dawley rats (250–280 g) were randomly allocated to receive either vehicle or one of three doses (n = 5 per group). Within each study, the operator was blinded to the treatment. Treatment was given for 7 days, via oral gavage according to the following scheme. Dimethadione study: vehicle (saline), 10, 30 or 100 mg/kg once daily; salbutamol study: vehicle (saline), 0.5, 5 or 50 mg/kg twice daily; trazodone study: vehicle (saline), 10, 30 or 50 mg/kg twice daily; trifusal study: vehicle (7.2 mM HNaCO_3_; pH7.4), 3, 10 or 30 mg/kg once daily. The dose ranges were determined by cross-analysis of existing literature, toxicity and pharmacokinetic data.

Within 2 h of the last dose, animals were killed by overdose of sodium pentobarbital (200 mg, i.p.) and the brain rapidly removed. The VM (containing the substantia nigra (SN)) and the striatum were dissected out and snap-frozen on dry ice. Tissues were then homogenised on ice, via high frequency sonication in 500 µl RIPA lysis buffer (Thermo Fisher) containing protease and phosphatase inhibitors (Roche). Neat homogenates were centrifuged (10,000 rpm) at 4 °C for 5 min before measurement of supernatant protein content via BCA assay. Samples were diluted to 1 mg/ml protein with dH_2_O before determination of FGF20 protein levels via ELISA, as described above.

### Examination of the neuroprotective potential of two FGF20 boosting drugs in the 6-OHDA partial lesion rat model of Parkinson’s disease

#### Drug treatment and 6-OHDA lesioning

The two most promising drugs from the *in vivo* screen (salbutamol and triflusal) were taken through to this preliminary neuroprotective study. A total of 40 rats were randomly assigned to four treatment groups (n = 10 per group). Rats were then dosed by oral gavage in an operator-blinded manner either twice-daily (9 am and 6 pm) with salbutamol (50 mg/kg) or salbutamol vehicle or once-daily (9 am) with triflusal (10 mg/kg) or triflusal vehicle. Doses were selected from the dose-range study, above. Dosing commenced (in a randomised block design to reduce daily bias between treatment groups) 2 days before induction of a partial 6-OHDA-induced lesion and continued daily for 7 days post-lesion. This dosing regimen was selected to maximise our chances of achieving neuroprotection by priming enhanced FGF20 production prior to introduction of the toxin as well as driving production during lesion development. For 6-OHDA lesioning, following anaesthesia induction (5% isofluorane/oxygen), animals were placed in a stereotaxic frame with blunt ear bars. Anaesthesia was maintained at 3% isofluorane/oxygen and body temperature maintained at 37 °C. The surgical site was sterilised with 0.4% chlorhexidine (Hibiscrub) before making an antero-proximal incision along the scalp. Fine-bore holes (Ø 0.5 mm) were made in the skull at coordinates AP: +0.2 mm and ML: +3.0 mm (relative to bregma and skull surface) through which a blunt-ended 30-gauge needle was inserted to DV: −5.5 mm. 6-OHDA.HBr (12 μg in 3 μl 0.02% ascorbate/saline) was infused unilaterally into the striatum (0.5 μl/min) and the needle withdrawn 5 min later. After suturing, animals received a single dose of buprenorphine (Vetergesic; 0.1 mg/kg; s.c.) for analgesia and 1 ml of rehydrating Hartmann’s solution was administered s.c. daily for 7 days. Two animals, later identified as allocated to the triflusal group, failed to recover adequately from surgery so were excluded from the study.

#### Immunohistochemical assessment of SNc lesion size

On post-lesion day 7, animals were killed (2 h post final drug treatment) with an overdose of anaesthetic (200 mg sodium pentobarbital) before removal of the brains. The mid-brain containing the SN was submerged in 4% paraformaldehyde for 24 hours. Fixed brains were then embedded in paraffin wax. Serial 7 µm sections of the SN were obtained and processed for tyrosine hydroxylase (TH) staining as previously described^7^. Photomicrographs of TH-immunostained SNc sections (n = 3 per rat at each of the caudal [AP: −6.0 mm], medial [AP: −5.3 mm] and rostral [AP: −4.8 mm] levels) were acquired at 20× magnification using a Zeiss fluorescent microscope and Axiovision software (Carl Zeiss Ltd). ImageJ software was used to manually count viable (i.e. intact round cells with a clear nucleus and cytoplasm) TH-positive A9 dopaminergic cells of the SNc in both the lesioned and intact hemispheres. SNc cell number in the lesion hemisphere was calculated a percentage of that remaining in the intact hemisphere. Data were combined across all three rostro-caudal levels to generate a single average value for each animal. Mean data were then calculated per treatment group.

### Statistical analyses

All statistical analyses were performed using GraphPad Prism (version 7). FGF20 protein levels in MCF-7 cells were compared between each drug and respective vehicle using a Student’s *t*-test. FGF20 protein levels in VM cell lysates were compared between each drug and shared vehicle using a one-way ANOVA with Dunnett’s post-hoc test. Similarly, FGF20 levels in striatal or VM homogenates from rat brain were compared between any dose of drug and vehicle using a one-way ANOVA with Dunnett’s post-hoc test. Differences in nigral TH-positive cell counts were compared between drug and vehicle-treated animals using Student’s *t*-tests.

## Results

### Bioinformatics screening to draw up shortlist of potential FGF20 boosting drugs

Of the top 50 ranking drugs identified through interrogation of the CMap database to increase FGF20 gene transcription, a number were eliminated due to low BBB penetration probability (e.g. sulconazole, cefsulodin and ketanserin). Other drugs were eliminated owing to contraindications for use in PD (e.g. antipsychotic drugs, pimozide and fluspirilene) or known toxicity associated with chronic use (e.g. flunixin) (listed in Supplementary Table S1). Sixteen drugs of interest remained: these came from a wide range of pharmacological classes and are, or have been, used for many different conditions (Table 1). All sixteen were taken forward into *in-vitro* investigations of FGF20 protein production.

**Table 1:** Candidate drugs for the up-regulation of FGF20.

Candidate	CMap rank	Expression rank	Class
dimethadione	2	0.74	anti-epileptic
atenolol	3	0.71	cardioselective β blocker
propranolol	5	0.69	non-selective β blocker
clonidine	6	0.67	α_2_ receptor agonist
estriol	9	0.65	estrogen
luteolin	10	0.65	flavonoid
salbutamol	18	0.58	β_2_ receptor agonist
equilin	19	0.58	horse estrogen
ethaverine	21	0.56	L-type Ca^2+^ channel blocker
torsemide	28	0.49	diuretic
sitosterol	30	0.45	phytosterol
levonorgestrel	33	0.42	oral contraceptive
triflusal	34	0.42	platelet aggregation inhibitor
trazodone	35	0.40	5HT_2A_ antidepressant and α_1_ receptor antagonist
betamethasone	38	0.38	steroid
testosterone	42	0.35	primary male sex hormone

The candidate drugs are ranked based on the expression levels across the 1261 drugs in the CMap database. The relative expression rank of FGF20 in the individual CMap expression profiles is shown in the third column. The remaining 26 drugs within the top 42 were dropped from further analysis due to their inability for blood-brain barrier penetration and having anticipated contraindications either when administered chronically or with other medications frequently used by people with Parkinson’s disease (see Supplementary Table S1).

### Effects of shortlisted drugs on FGF20 production in MCF-7 cells and VM primary cultures

Absolute basal levels of FGF20 varied between MCF-7 cell cultures, ranging in vehicle-treated groups from 8 to 28 pg/ml. For this reason, FGF20 protein levels were standardised in vehicle-treatment groups between cultures and the drug effects expressed thereafter as fold-increase in expression. When testing the 16 drugs on MCF-7 cells, 11 significantly elevated FGF20 protein levels compared to their respective vehicles (Table 2): the four most significant (dimethadione, salbutamol, trazodone and triflusal) were selected for further testing on primary VM cultures. Although propranolol looked promising in terms of significance of FGF20 protein elevation, it was omitted from further examination at this stage in light of emerging data linking its use to the enhanced risk of PD^19^.

**Table 2:** FGF20 levels following 10 µM drug treatment in MCF-7 cells.

Candidate	FGF20 fold increase	SEM	*P* value	
**dimethadione**	4.40	0.87	0.0095	**
**trazodone**	2.68	0.23	0.0024	**
**salbutamol**	2.69	0.13	0.0002	***
atenolol	2.92	0.49	0.013	*
propranolol	4.29	0.50	0.002	**
levonorgestrel	3.38	1.03	0.067	
sitosterol	2.19	0.93	0.189	
clonidine	2.99	0.71	0.013	*
luteolin	2.06	0.32	0.019	*
torsemide	3.92	1.20	0.036	*
estriol	2.09	0.41	0.05	
**triflusal**	3.72	0.78	0.003	**
ethaverine	3.65	0.66	0.013	*
betamethasone	2.29	0.81	0.08	
testosterone	2.57	0.48	0.016	*
equilin	2.39	0.76	0.16	

Drugs in bold were taken forward into ventral mesencephalic primary culture studies. *P* value obtained by Student’s *t*-test analysis between drug treatment and individual vehicle controls. *^,^ ** and *** are *P* values < 0.05, <0.01 and <0.001, respectively.

**Figure 1:**
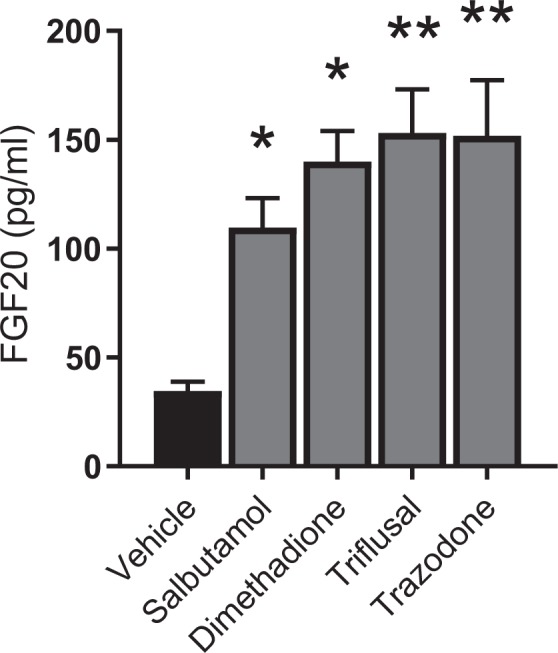
FGF20 production in ventral mesencephalic cultures following 24 h incubation with candidate drug (@10 µM). Data are mean ± SEM. n = 3 litters per treatment. *P < 0.05; **P < 0.01 versus vehicle treatment (ANOVA with Dunnett’s post hoc test).

All four selected drugs significantly elevated FGF20 levels in VM cultures compared to vehicle (Fig. 1). Basal FGF20 levels obtained from the VM culture screens were more consistent between cultures therefore data could be represented as raw values. Fold-changes were of similar magnitude to those seen in MCF-7 cells: between 2 and 4-fold increases compared to vehicle. Although not quantified, visual inspection of the cells prior to lysis, revealed healthy looking cells, with no obvious signs of loss of cell viability. All four drugs were accordingly taken through for *in-vivo* examination of FGF20 production.

### Effects of selected drugs on FGF20 production in naïve rats

**Figure 2:**
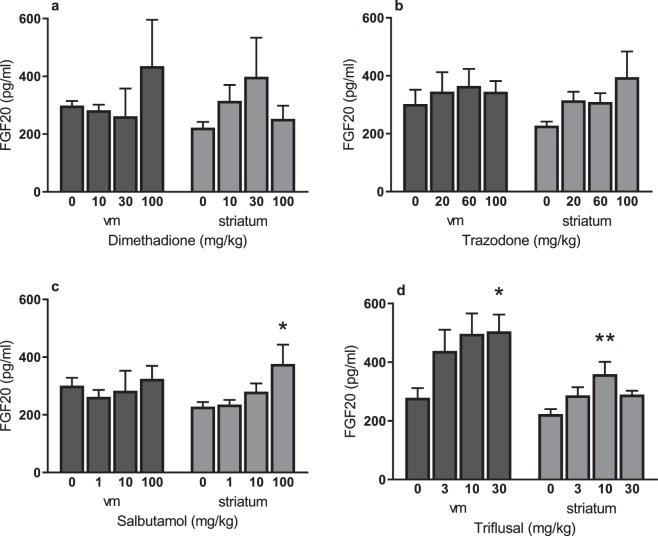
Quantification by ELISA of FGF20 production in the ventral mesencephalon (vm) and striatum of adult male rats dosed for 7 days with dimethadione (**a**), trazodone (**b**), salbutamol (**c**), triflusal (**d**) or respective vehicle (0 = saline). Data are mean ± SEM. n = 5 per dose. *P < 0.05; **P < 0.01 versus vehicle (ANOVA with Dunnett’s post hoc test).

Although dimethadione displayed a trend towards increasing FGF20 within the striatum and VM, neither it nor trazodone significantly elevated endogenous FGF20 in either the striatum or VM following 7 days’ dosing in rats (Fig. 2a,b). In contrast, salbutamol (100 mg/kg) significantly increased striatal endogenous FGF20 levels 1.7-fold over vehicle treatment (one-way ANOVA with Dunnett’s post hoc test, p = 0.037; Fig. 2c). Triflusal (10 mg/kg) similarly increased striatal FGF20 1.6-fold over vehicle treatment; one-way ANOVA with Dunnett’s post hoc test, p = 0.008), but also produced a significant increase within the VM (1.8-fold over vehicle treatment; one-way ANOVA with Dunnett’s post hoc test, p = 0.044) at 30 mg/kg (Fig. 2d). Having confirmed the ability of salbutamol and triflusal to significantly elevate FGF20 levels *in vivo*, these drugs were next examined for neuroprotective potential.

### Preliminary examination of the neuroprotective potential of the FGF20 boosting drugs, salbutamol and triflusal, in unilaterally 6-OHDA-lesioned rats

**Figure 3:**
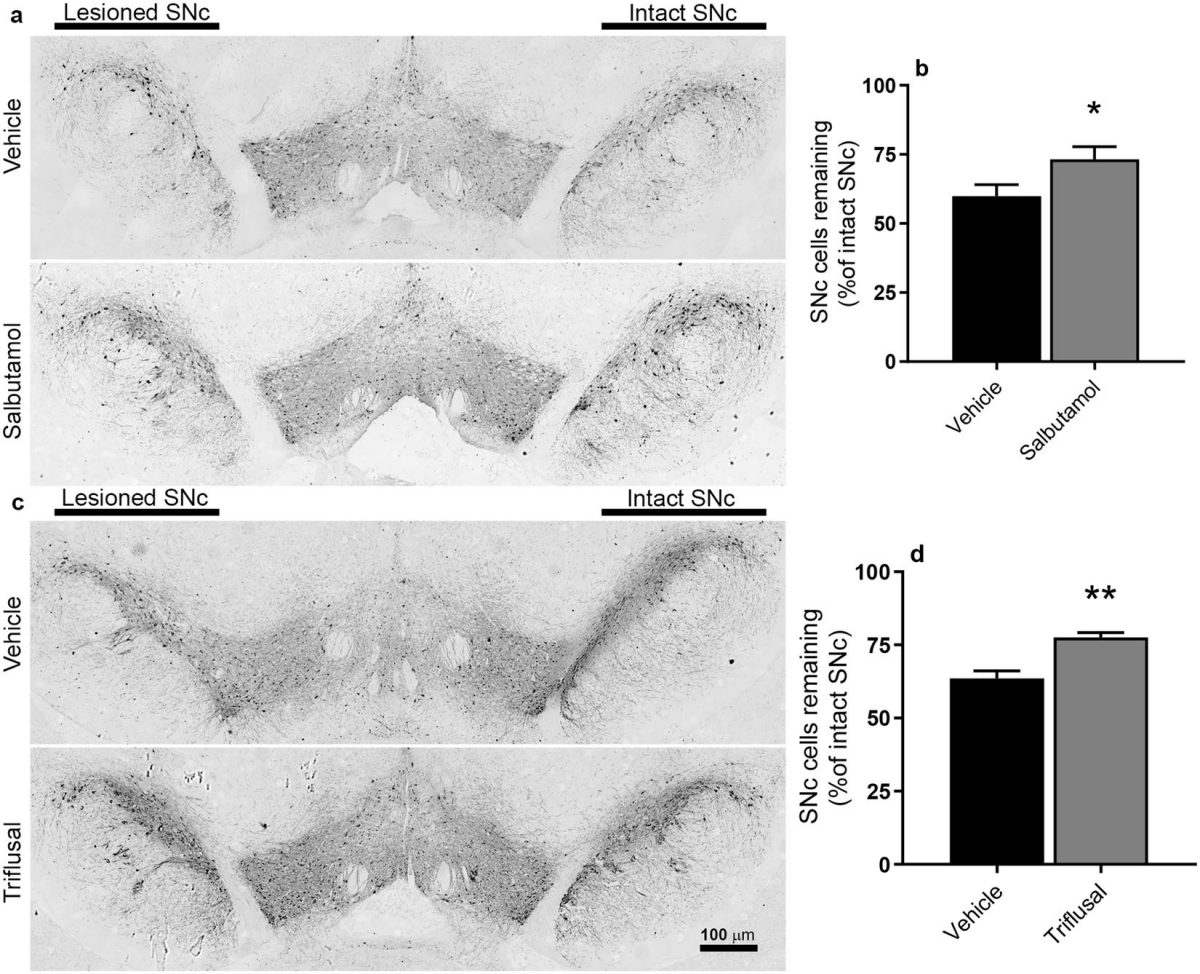
Neuroprotective effects of salbutamol (50 mg/kg, twice-daily for 9 days) or triflusal (10 mg/kg, once-daily for 9 days) in unilaterally 6-OHDA-lesioned rats. (**a**,**c**) Photomicrographs displaying the TH-positive SNc cells within the lesioned (left) and intact sides of drug and respective vehicle-treated controls. Quantification of SNc cells reveals significant protection in rats treated with salbutamol (**b**) or triflusal (**d**). *P < 0.05; **P < 0.01 (Student’s *t*-test). Data are mean ± SEM. n = 10 for all groups except triflusal (n = 8).

Both salbutamol and triflusal displayed neuroprotective efficacy in this partial 6-OHDA-lesioned rat model of PD. Representative photomicrographs taken from the medial level of the SNc show increased cells in the lesion hemisphere from salbutamol-treated (Fig. 3a) or triflusal-treated (Fig. 3c) animals when compared to their respective vehicle-treated controls. Although manual rather than the more comprehensive stereological analysis was performed, studies comparing these two modes of counting in the 6-OHDA model have found no significant difference in the outcome between these measures, supporting the validity of our analysis^20^. Furthermore, the mean number of cells counted in the intact hemisphere per section was consistent with previous reports by this and other groups of around 100 cells^20,21^: 109.4 ± 2.6 in salbutamol vehicle group; intact: 104.0 ± 6.5 in salbutamol treatment group; 100.6 ± 8.7 in triflusal vehicle group; 97.3 ± 8.2 in triflusal treatment group. Across the extent of the SNc, salbutamol increased the percentage of SNc cells remaining in the lesioned hemisphere relative to the intact hemisphere from 59.9% ± 4.1 (n = 10) in vehicle-treated animals to 73% ± 4.5 (n = 10) (p < 0.05; Fig. 3b). Similarly, triflusal administration increased SNc cells in the lesioned hemisphere from 63.5% ± 2.6 of intact hemisphere in vehicle-treated animals (n = 10) to 77.6% ± 1.6 (n = 8) (p < 0.01; Fig. 3d).

## Discussion

This study set out to explore whether existing drugs could be used to increase endogenous FGF20 production in the nigrostriatal tract, rather than relying on direct intracerebral infusion of recombinant FGF20. To do so we employed a *targeted* repositioning strategy, a top-down method whereby the target protein of interest was firstly identified, in this case FGF20, before subsequent computational, transcriptomic and literature-based mining was used to generate a shortlist of compounds. Protein quantification assays were then conducted to further filter out candidates that did not increase endogenous FGF20 levels before, finally, candidates were tested in a small-scale neuroprotection study. Results of this final stage revealed two drugs worthy of future investigation as neuroprotective agents in PD – salbutamol and triflusal.

The targeted repositioning strategy was conducted in a step-wise manner, starting with *in silico* screening to interrogate the CMap transcriptional database. Using the SPIEDw algorithm^18,22^, a long-list of 50 FDA-approved drugs boosting FGF20 gene transcription in human breast carcinoma cells was obtained. Following literature-based mining to identify and hence remove any compounds that were known to not cross the BBB, present toxicity following chronic use or be contraindicated for use in people with Parkinson’s, 16 drugs of interest remained. These were screened for the ability to boost FGF20 protein production *in vitro*. Although the initial MCF-7 cell line of choice bears no resemblance to PD-relevant cells, their use was deliberate for two reasons. Firstly, since these cells had been used to populate the CMap transcriptional database, discovering that 11 of the 16 drugs tested significantly elevated FGF20 production in these cells (between 2 and 4.5-fold), generated confidence in the translational potential between the gene expression changes and accomplished protein production. Secondly, selecting those drugs that proved most effective in the MCF-7 cells, enabled restriction of the number that progressed through to primary cultures, thereby ensuring NC3R compliance.

All four drugs taken into VM cultures increased FGF20 production. These mixed neuronal and glial cultures were ideal for our purposes, since FGF20 appears to be made not in dopaminergic neurones themselves, but rather in astrocytes and other, as yet unidentified cells^7^. The efficacy of the drugs was similar in VM cultures as was observed in the MCF-7 cells in terms of fold-increase in FGF20 levels, suggesting the molecular mechanisms underpinning the increased transcription and translation are likely similar between the two different cell systems.

Given the significant increases noted in the VM cultures, all four drugs progressed through to *in vivo* examination. Doses selected for investigation were in line with those used in previous unrelated rodent studies and fell significantly below known LD50 values^23–26^. Although both dimethadione and trazodone produced some elevation in FGF20 protein levels, these failed to reach significance. While they were not taken further in the present study, given the strength of their effects *in vitro*, these drugs remain of interest. Indeed, these initial *in vivo* studies were conducted with small sample sizes, so false negatives cannot be ruled out. Salbutamol and triflusal both significantly elevated FGF20 protein levels. Salbutamol increased FGF20 in the striatum but not within the VM, while triflusal increased FGF20 in both the striatum and VM (containing SNc). Given we have demonstrated FGFR1, 3 and 4 expression on TH-positive cells in the SNc and FGFR1 expression on striatal TH-positive terminals^7^, elevating FGF20 levels in either the striatum or the SNc has the potential to impact on the survival of dopaminergic neurones. The cellular source of elevated FGF20 is still subject to ongoing investigation. Astrocytes have already been identified as a key source in the SNc^7^ and our recent findings support astrocytes being a source of FGF20 in the striatum too (see Supplementary Fig. S2). Nevertheless, FGF20 co-localises with at least one other type of non-dopaminergic cell^7^, that could act as an additional, potential source of production and ongoing studies will help to identify this.

Of note, there was a decline in striatal FGF20 production at the highest doses of triflusal. The reason behind this remains to be determined but the possibility of receptor desensitisation will need to be considered when further exploring the effective dose range.

As a final check on the validity of this repositioning process to locate drugs of interest to provide neuroprotection in PD, a small-scale neuroprotection study was conducted. Following a week of treatment, both salbutamol and triflusal were able to provide modest, but significant protection to SNc cells in a 6-OHDA rat model of PD. Given the mild size of lesion, we were not able to examine for behavioural improvements using cylinder and adjusted stepping tests. This is something that will require addressing in the future in animals bearing a larger nigrostriatal tract lesion in order to confirm functionality of the protected neurones.

Since commencing this work in early 2017, two of the four candidates that progressed into our *in vivo* stage have been published as contenders for therapies targeting neurodegenerative diseases. The first of these is the β-adrenergic receptor agonist salbutamol^19^, a commonly used drug for asthma and COPD. Mittal *et al*. suggest salbutamol’s protective mechanism relates to the downregulation of SNCA gene transcription and that this is directly related to the adrenergic activity of the compound. Mittal *et al*. further report that salbutamol lowers risk of PD incidence in a large-scale epidemiological analysis of a Norwegian population whilst propranolol, a non-selective β-adrenergic receptor antagonist, increases risk (hence it being withdrawn from further investigation in the present study). Similar benefits and risks of salbutamol and propranolol have been recently supported by some^27^, but not other^28^ independent reports. Although we too suggest salbutamol as a potential protective therapy for PD, we have identified a distinct mechanism, that of increased endogenous FGF20 transcription within PD-relevant tissues. We propose these two mechanisms may work together to provide neuroprotection in PD, although further studies utilising FGFR antagonists are required to confirm the involvement of FGF20 in salbutamol’s action.

The second drug highlighted by others is the antidepressant, trazodone^29^. Halliday *et al*. identified trazodone as a drug that would reduce the phosphorylated alpha-subunit of eukaryotic initiation factor 2 (eIF2α-P), an initiation factor found at high levels within the brains of people with PD and other neurodegenerative diseases^30^. Its presence is linked with the deposition of phosphorylated tau^31^ and its over activation has shown to cause degeneration and synapse degradation^32^. Interestingly trazodone presents α1- and α2-adrenergic receptor antagonism^33^, highlighting the potential interaction of the adrenergic system and neuroprotection. Although trazodone enhanced FGF20 production within our cell screens it did not within our *in vivo* tests. It will therefore be of interest to understand whether trazodone is neuroprotective through this eIF2α-P mechanism, and whether trazodone retains efficacy in the proposed future human trials.

To summarise, we previously reported that direct intracerebral infusion of FGF20 is neuroprotective in the partial and full 6-OHDA lesioned rat model of PD^6^. We now demonstrate that we can boost endogenous brain levels of FGF20 using drugs originally identified from *in silico* screening of transcriptional databases and subsequently screened through a reductionist approach *in vitro* and then *in vivo*. Two of these drugs, salbutamol and triflusal, afforded significant protection against 6-OHDA lesioning in a mild 6-OHDA lesion model of PD. Current studies are underway to more thoroughly investigate the neuroprotective potential of salbutamol and triflusal and to identify the potential mechanisms involved in enhancing FGF20 gene transcription.

In conclusion, the work presented here highlights the potential of *targeted repositioning* as a valid strategy to identify drugs that can affect target-gene transcription and, ultimately, protein production in PD. By adopting such a repositioning strategy, any promising drugs identified have the potential to progress into clinical trials with a shorter lead in time given the safety and tolerability is already established. Having validated this targeted approach, the opportunity now exists to apply the strategy to other proteins of interest in PD and beyond.

## Supplementary information

Supplementary Information File

## Data Availability

All data generated or analysed during this study are included in this published article (and its Supplementary Information files) or available from the corresponding author on reasonable request.
