# Supplementary material for: Targeted repositioning identifies drugs that increase fibroblast growth factor 20 production and protect against 6-hydroxydopamine-induced nigral cell loss in rats
Source: Sci Rep. 2019 Jun 6;9:8336. doi: 10.1038/s41598-019-44803-1 (PMC6554393; doi:10.1038/s41598-019-44803-1)
Supplement: Supplementary file 1 — Supplementary Information File [file 41598_2019_44803_MOESM1_ESM.pdf]

Supplementary Information File

Targeted repositioning identifies drugs that increase fibroblast growth factor 20 production and protect against 6-hydroxydopamine-induced nigral cell loss in rats.

Edward J R Fletcher, Aran D Jamieson, Gareth Williams, Patrick Doherty and Susan Duty.

Supplementary Table S1. Candidate drugs from the *in-silico* screen that did not progress to *in-vitro* screening.

Candidate	CMap rank	Expression rank	Class
6-azathymine	1	0.85	anti-cancer compound
cefsulodin	4	0.7	cephalosporin antibiotic
ginkgolide A	7	0.66	natural product; platelet aggregation inhibitor
gramine	8	0.66	naturally occurring indole alkaloid; NA reuptake inhibitor
adipiodone	11	0.63	contrast medium
corynanthine	12	0.63	natural alkaloid
cefalotin	13	0.62	cephalosporin antibiotic
rimexolone	14	0.6	glucocorticoid
flunixin	15	0.59	veterinary NSAID
sulconazole	16	0.59	antifungal
clomipramine	17	0.58	tricyclic antidepressant
gelsemine	20	0.57	natural alkaloid
omeprazole	22	0.55	proton pump inhibitor, gastric anti-acid
benzylpenicillin	23	0.53	penicillin G
methylbenzethonium chloride	24	0.53	antiseptic
thiabendazole	25	0.51	fungicide, anthelmintic
fluspirilene	26	0.5	typical antipsychotic
hecogenin	27	0.49	plant steroid
ketanserin	29	0.46	antihypertensive
dacarbazine	31	0.44	antineoplastic chemotherapeutic
pimozide	32	0.43	typical antipsychotic
0175029-0000	36	0.39	cyclin-dependent kinase inhibitor
meclozine	37	0.39	antihistamine
alexidine	39	0.37	antimicrobial
meticrane	40	0.36	diuretic
bisacodyl	41	0.35	laxative

The candidates are ranked based on the expression levels across the 1261 drugs in the CMap database. The relative expression rank of FGF20 in the individual CMap expression profiles are shown in the third column. All these drugs were rejected prior to *in-vitro* screening based on their inability to cross the blood-brain barrier or because they possessed contraindications or other undesirable side-effects for people with Parkinson’s disease.

Supplementary Figure S2. Astrocytes are a source of FGF20 production in the rat striatum

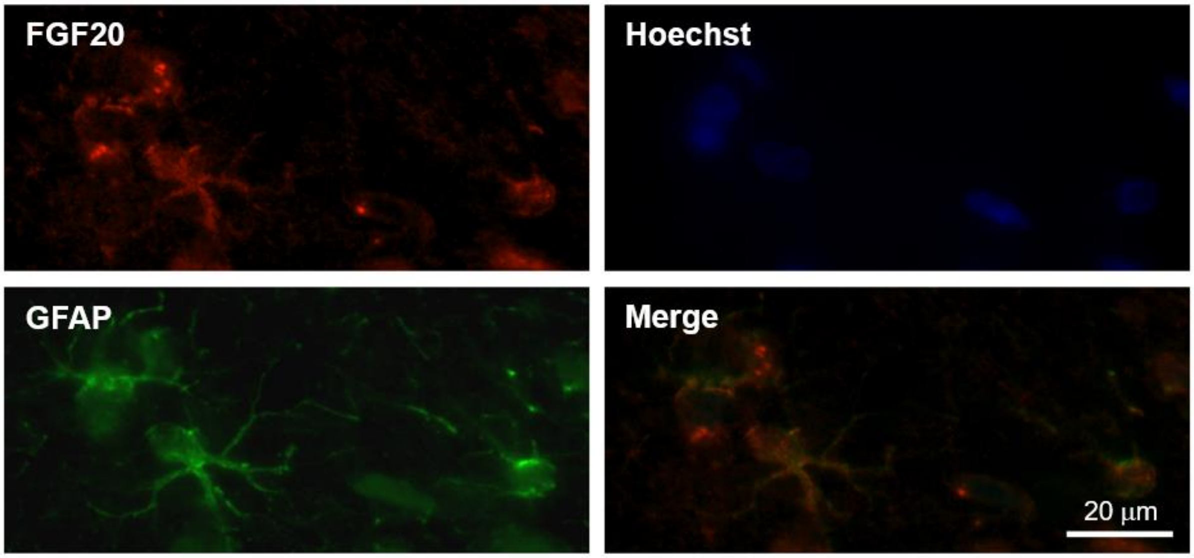

Fibroblast growth factor 20 (FGF20) co-localises with glial fibrillary acidic protein (GFAP) in naïve rat striatum indicating astrocytes as a source of FGF20 in this region.

Paraffin-embedded naïve adult rat brain sections (8 µm) were incubated with bovine serum albumin blocking buffer for 10min before being incubated with rabbit polyclonal anti-FGF20 primary antibody (abcam, ab198876, 1/200) and mouse polyclonal anti-GFAP primary antibody (abcam, ab7260, 1/500) overnight. Following TBS washes, the sections were incubated for 1 hour with goat anti-rabbit AlexaFluor 594 (1/750) and goat anti-mouse AlexaFluor 488 (1/750). Nuclei were stained with hoechst 33342. All steps were carried our at room temperature.

Images were obtained at 20x magnification using a Zeiss Apotome fluorescent microscope and Axiovision software.
